# ^210^Po microsphere radiological design for tumor vascular disruption

**DOI:** 10.7717/peerj.1143

**Published:** 2015-08-04

**Authors:** JJ Bevelacqua

**Affiliations:** Bevelacqua Resources, Richland, WA, USA

**Keywords:** Absorbed dose, Dose assessment, Tumor vascular disruption, Radiation therapy, Microspheres

## Abstract

The feasibility of disrupting a tumor’s vascular structure using ^210^Po microspheres is investigated using standard ion and photon absorbed dose methodologies. Calculated absorbed dose profiles for ^210^Po alpha particles are sufficient to disrupt a tumor’s arteriole structure while minimizing the dose outside the blood vessel wall. ^210^Po photons contribute minimal dose to healthy tissue. The requisite activity of ^210^Po to facilitate vascular disruption is calculated.

## Introduction

A significant issue associated with existing radiotherapy approaches is that agents that deliver dose to tumor cells also irradiate healthy tissue. The irradiation of healthy tissue following the administration of therapeutic radionuclides affects the patient’s quality of life and leads to short-term as well as long-term detriments. Although the short-term detriment varies with the specific therapy approach, it is illustrated with radiotherapy methods for prostate cancer. The short-term effects include incontinence and erectile dysfunction that affects the patient’s recovery and subsequent quality of life (Martinez et al., 2000). Long-term effects of radiotherapy can include secondary cancers and cardiovascular disease (NCRP, 2012). For these reasons, it is important to continue to investigate alternative radiotherapy approaches that deliver dose selectively to the target tissue (i.e., tumor mass or vascular structure) and minimize dose to healthy tissue.

A unique approach that has the potential to impact tumor cells involves disrupting its vascular structure. A number of authors (Folkman, 1971; Burke & DeNardo, 2001) have proposed the basis for a therapy approach that prevents the development of the tumor’s vascular supply. Vascular disruption agents incorporate both chemotherapy (Carmeliet & Jain, 2000; Rajput & Agrawal, 2010) as well as radiotherapy (Kennedy & Salem, 2003; Carr, 2004; Murthy et al., 2005; Kennedy et al., 2007; Chaudhury et al., 2010; Rajput & Agrawal, 2010; Dezarn et al., 2011; Lau, Lai & Leung, 2011; Lee & Seong, 2012; Amor-Coarasa et al., 2014). These approaches are also known as anti-angiogenic or radioembolization therapies.

Radiotherapy vascular disruption techniques have been extensively applied to liver cancers (Kennedy & Salem, 2003; Carr, 2004; Murthy et al., 2005; Kennedy et al., 2007; Chaudhury et al., 2010; Rajput & Agrawal, 2010) utilizing ^90^Y microspheres. Other radionuclides (e.g., ^32^P) have been less thoroughly investigated and radiation types other than high-energy beta particles have not been systematically investigated (Rajput & Agrawal, 2010).

In a previous paper (Bevelacqua, 2014), candidate microsphere characteristics for disrupting a tumor’s vascular structure were evaluated. The goal of this initial paper was to investigate the characteristics of various radiation types to minimize the absorbed dose delivered beyond the vascular wall and to limit the dose delivered to healthy tissue. This stray dose is encountered in currently available microspheres incorporating ^90^Y and ^32^P (Kennedy & Salem, 2003; Carr, 2004; Murthy et al., 2005; Kennedy et al., 2007; Chaudhury et al., 2010; Rajput & Agrawal, 2010; Dezarn et al., 2011; Lau, Lai & Leung, 2011; Lee & Seong, 2012; Amor-Coarasa et al., 2014). Unlike the ^90^Y approach that has been successfully applied in a clinical setting, the proposed approach is theoretical and has yet to be utilized in treatment applications.

Theoretical dose localization was obtained with internal radiation-generating devices (Bevelacqua, 2005; Bevelacqua, 2010a; Bevelacqua, 2012) using 4–7 MeV alpha particle beams (Bevelacqua, 2014). Since these devices are theoretical, various radionuclides were evaluated (Bevelacqua, 2014), but most alpha particle emitting radionuclides are associated with decay chains that have undesirable beta and gamma radiation types. Unfortunately, this beta-gamma radiation has sufficient energy to irradiate healthy tissue, and partially negates the beneficial effect of the alpha radiation.

This paper advances the work of Bevelacqua (2014) and selects a candidate microsphere radionuclide for vascular disruption that facilitates dose localization within an arteriole wall. Dose localization is facilitated using ^210^Po since it is near the end of the ^222^Rn decay chain, and it has a low yield gamma-ray that minimizes the dose delivered beyond the tumor vascular wall.

## Tumor Vasculature

Solid tumors have a unique vascular configuration (Carmeliet & Jain, 2000). In normal tissues, the vasculature structure is arranged to provide optimum nourishment conditions. In general, growing tumors have a chaotic vasculature that is not fully developed or adequate to optimally nourish the tumor cells. Since a tumor’s growth is dependent on sufficient nourishment, its viability is affected by disrupting the blood supply. In principle, eliminating a tumor’s blood supply provides an alternative therapy approach to facilitate or supplement its destruction.

## Current Radiological Efforts

Radiological efforts at tumor vascular disruption have focused on ^90^Y. ^90^Y was a logical choice for anti-angiogenic therapy since the dose (≥70 Gy) for the vascular disruption of a tumor (Kennedy et al., 2007) is easily achieved. Most of this effort focuses on a variety of liver carcinomas. However, the ^90^Y beta particles have significant range and extend well beyond the vascular target. The maximum ^90^Y beta energy of 2.27 MeV has a range in tissue of about 1.1 cm, which delivers absorbed dose beyond the target vascular structure. Additional stray dose is delivered by the associated ^90^Y bremsstrahlung radiation.

Commercially available ^90^Y microsphere properties are summarized in Table 1. A discussion of resin and glass microspheres is provided by Bilbao & Reiser (2008) and Kennedy & Salem (2003), Carr (2004), respectively.

**Table 1:** Properties of resin and glass ^90^Y microspheres.

Parameter	Microsphere type	
	Resin	Glass
Diameter (µm)	20–60	20–30
Density (g/cm^3^)	1.6	3.6
Activity per microsphere (Bq)	50	2,500
Number of microspheres per 3 GBq vial (×10^6^)	40–80	1.2
^90^Y form	Yttrium bound to resin	Yttrium in glass matrix
Derived from Kennedy et al. (2007)		

## Proposed Treatment Methodology

Conventional radiotherapy attacks a tumor mass using external beams or radionuclides (Kraft, 2000; Bevelacqua, 2010a). The proposed work follows the ^90^Y vascular disruption approach (Kennedy & Salem, 2003; Carr, 2004; Murthy et al., 2005; Kennedy et al., 2007; Chaudhury et al., 2010; Rajput & Agrawal, 2010; Dezarn et al., 2011; Lau, Lai & Leung, 2011; Lee & Seong, 2012; Amor-Coarasa et al., 2014) to disrupt the tumor blood supply. This action starves the tumor of nutrients and has been effectively applied to liver tumor treatments using ^90^Y microspheres. Eliminating the supply of nutrients to the tumor is an alternative method to attack this growth and terminate its viability. However, the ^90^Y microsphere approach delivers dose well beyond the vascular wall.

The ^210^Po microsphere approach refines the ^90^Y methodology to deliver dose preferentially to vascular structures. As such, absorbed dose is delivered to the vascular wall to damage its structure and restrict nutrient delivery to the tumor. This paper selects the arteriole as the target tissue. As will be noted in subsequent discussion, the ^210^Po alpha particle delivers a disruptive dose to a range of 37 µm in water which is sufficient to cause damage throughout the arteriole wall. Given the current stage of development, water has been selected as a surrogate for tissue. This approach has been utilized by other authors in their initial calculational efforts to develop therapy approaches (Kraft, 2000). Details of implanting the ^210^Po microspheres in tissue, associated delivery methods, and other required therapy protocol actions are addressed in subsequent discussion.

## Methods

Alternatives to ^90^Y microspheres are investigated by extending the search for alternative nuclides to load a vascular disruption microsphere (Bevelacqua, 2014). This is accomplished by selecting a radionuclide and its associated daughters that preferentially deliver absorbed dose to the vascular wall.

### Experimental approach

The proposed ^210^Po microsphere approach is currently limited to a theoretical treatment. This is expected since the initial investigation (Bevelacqua, 2014) determined the appropriate radionuclide characteristics, radiation types, and associated energies to facilitate vascular disruption. Bevelacqua (2014) outlined these global parameters, which is the first step in developing the proposed microsphere vascular disruption therapy approach. The next step, outlined in this paper, is the selection of a candidate radionuclide to load the microsphere and determination of its absorbed dose deposition profile. Detailed design in terms of the specific materials of construction, optimum size and shape, and radionuclide distribution within the microsphere to optimize the deposition characteristics within the target blood vessel will be addressed in subsequent research. Experimental work would follow the completion of the aforementioned tasks and construction of the designed microspheres loaded with candidate radioactive material.

These theoretical steps are necessary to develop the requisite parameters to determine a viable experimental approach. Accordingly, no experimental results are provided in this paper.

This situation is similar to the development of an antiproton therapy modality (Holzscheiter et al., 2006; Bittner et al., 2013). Theoretical calculations were the initial steps in establishing the feasibility of the antiproton therapy method. These calculations required a number of years to refine and establish the appropriate parameters for the design of an experimental approach.

## Selection of Radionuclide

In the initial vascular disruption paper (Bevelacqua, 2014), a list of candidate radionuclides was created with a goal of minimizing the dose delivered beyond the arteriole wall. As part of that goal, ^222^Rn was noted as an interesting possibility since it occurs naturally as part of the ^238^U decay chain. Table 2 lists the ^222^Rn decay daughters and their characteristic radiation types with the associated decay scheme:

(1) \begin{eqnarray*} \displaystyle {\text{}}^{222}\mathrm{Rn}\hspace{0.167em} \mathop { \rightarrow }\limits ^{\alpha }\hspace{0.167em} {\text{}}^{218}\mathrm{Po}\hspace{0.167em} \mathop { \rightarrow }\limits ^{\alpha }\hspace{0.167em} {\text{}}^{214}\mathrm{Pb}\hspace{0.167em} \mathop { \rightarrow }\limits ^{{\beta }^{-}}\hspace{0.167em} {\text{}}^{214}\mathrm{Bi}\hspace{0.167em} \mathop { \rightarrow }\limits ^{{\beta }^{-}}\hspace{0.167em} {\text{}}^{214}\mathrm{Po}\hspace{0.167em} \mathop { \rightarrow }\limits ^{\alpha }\hspace{0.167em} {\text{}}^{210}\mathrm{Pb}\hspace{0.167em} \mathop { \rightarrow }\limits ^{{\beta }^{-}}\hspace{0.167em} {\text{}}^{210}\mathrm{Bi}\hspace{0.167em} \mathop { \rightarrow }\limits ^{{\beta }^{-}}\hspace{0.167em} {\text{}}^{210}\mathrm{Po}\hspace{0.167em} \mathop { \rightarrow }\limits ^{\alpha }\hspace{0.167em} {\text{}}^{206}\mathrm{Pb}.&&\displaystyle \end{eqnarray*}

**Table 2:** ^222^Rn and its daughters^a^^,^^b^^,^^c^.

		Radiation type/energy (MeV)		
Nuclide	*T*_1/2_	Alpha	Beta	Gamma
^222^Rn	3.8235 d	5.4895	—	0.510 (w)
^218^Po	3.10 min	6.0024	w	0.510
^214^Pb	27 min	—	0.67	0.3519
			0.73	0.2952
				0.2420
^214^Bi	19.9 min	5.450 (w)	3.27	0.6093
		5.513	1.54	1.7645
			1.51	1.1203
^214^Po	163.7 µs	7.6869	—	0.799 (w)
^210^Pb	22.3 y	3.72 (vw)	0.017	0.0465
			0.061	
^210^Bi	5.10 d	4.648 (vw)	1.162	0.305 (vw)
		4.687		0.266
^210^Po	138.38 d	5.3044	—	0.8031 (vw)
^206^Pb	Stable	–	—	—

**Notes.**

a Baum et al. (2010).

b Weak (w) transition with a yield <1%.

c Very weak (vw) transition with a yield <10^−3^%.

Although ^222^Rn is readily available, its daughters have beta and gamma radiation types that deliver significant absorbed dose well beyond the arteriole wall. Accordingly, it is not an optimum radionuclide to localize dose preferentially within the vascular wall. However, an examination of the ^222^Rn decay chain suggests that ^210^Po has the desired characteristics for vascular disruption without significantly irradiating healthy tissue. In particular, its 5.3 MeV alpha particle has sufficient energy to irradiate the arteriole wall while limiting absorbed dose beyond the target tissue. In addition, the weak 803 keV photon radiation with a yield of <1.0 × 10^−3^% delivers minimal dose beyond the target tissue.

### Theoretical approach

Disrupting a tumor’s vascular structure using radiotherapy could be performed as a stand-along protocol, the first step in a protocol followed by a chemical vascular disrupting agent (VDA), a final step in a therapy procedure initiated by a VDA, or part of an alternating sequence involving both radiotherapy and VDAs. This paper does not select a specific therapy approach, and only investigates the capability of ^210^Po loaded microspheres to disrupt an arteriole’s wall. As such, the present work is strictly theoretical, but offers the potential for enhancing existing tumor treatment approaches.

The blood supply to a tumor could be disrupted by damaging the vessel wall, causing the vessel to become restricted, or increasing its leakage. Table 3 summarizes the geometry of a variety of human blood vessel types (Barrett et al., 2012) including those that could service a developing tumor. A review of the literature suggests developing tumor vessel wall sizes will typically be less than 100 µm (Less et al., 1991; Barrett et al., 2012). This wall size includes arterioles, which are the assumed base case for the calculations presented in this paper.

**Table 3:** Characteristics of various blood vessel types.

Blood vessel type	Wall thickness	Lumen diameter
Aorta	2 mm	25 mm
Artery	1 mm	4 mm
Arteriole	20 µm	30 µm
Capillary	1 µm	8 µm
Venule	2 µm	20 µm
Vein	0.5 mm	5 mm
Vena cava	1.5 mm	30 mm
Derived from Barrett et al. (2012)		

### Base case microsphere design

The selected microsphere is loaded with ^210^Po with an activity of 0.3 Bq that is uniformly distributed in a 1 µm diameter sphere. The sphere is comprised of a ^12^C matrix with a density of 2.0 g/cm^3^. The basis for the 0.3 Bq ^210^Po microsphere activity is provided in subsequent discussion.

The calculations provided in the subsequent discussion are based on a single microsphere. Actual treatment protocols will entail a collection of these spheres with the number determined by the target cancer and its stage of development. Given the microsphere size, delivery methods other than the catheter approach (Bevelacqua, 2014) may be feasible. However, initial efforts will likely focus on the traditional catheter delivery approach (Carmeliet & Jain, 2000; Kennedy & Salem, 2003; Carr, 2004; Murthy et al., 2005; Kennedy et al., 2007; Chaudhury et al., 2010; Rajput & Agrawal, 2010).

### Absorbed dose computational model

As noted previously, an absorbed dose of about 100 Gy is sufficient to disrupt a tumor’s vasculature. An activity of 0.3 Bq of ^210^Po is sufficient to deliver an absorbed dose of about 100 Gy to the arteriole wall. No attempt has been to provide a more exact activity since the design has yet to be refined and must consider insertion and removal methods, fabrication details, and appropriate relative biological effectiveness values for the alpha and gamma ^210^Po radiation types.

### Alpha particle absorbed dose

For a tissue volume irradiated by ions of a given energy, the absorbed dose (*D*) as a function of penetration distance *x* into tissue is (Kraft, 2000; Bevelacqua, 2005):

(2) \begin{eqnarray*} \displaystyle D(x)=\frac{1}{\rho }\left(-\frac{d E}{d x}\right)\Phi (x)&&\displaystyle \end{eqnarray*}

where *ρ* is the density of the material (tissue, tumor, or other structure) attenuating the ion, −*dE*/*dx* is the stopping power, and Φ(*x*) is the ion fluence at the location of interest based on a 0.3 Bq ^210^Po microsphere. The particle fluence varies with tissue penetration depth according to the relationship:

(3) \begin{eqnarray*} \displaystyle \Phi (x)=\Phi (0){\mathrm{e}}^{-\Sigma x}&&\displaystyle \end{eqnarray*}

where Φ(0) is the entrance fluence and Σ is the macroscopic reaction cross-section.

Ion stopping powers are determined using Bethe’s formulation (Bethe, 1930) and follow an approach similar to SPAR (RSICC, 1985). The energy dependent cross-sections are obtained from Shen’s parameterization (Shen et al., 1989).

### Photon absorbed dose

The photon absorbed dose is derived from the standard point source relationships (Bevelacqua, 2010b)

(4) \begin{eqnarray*} \displaystyle D=\frac{S}{4 \pi {r}^{2}}\frac{{\mu }_{e n}}{\rho }E B(\mu x){\mathrm{e}}^{-\mu x}&&\displaystyle \end{eqnarray*}

where *S* is the total number of photons that irradiates the arteriole wall, *r* is the distance from the microsphere, *μ*_en_/*ρ* is the mass-energy absorption coefficient, *E* is the photon energy, *B* is a buildup factor, and *μ* is the attenuation coefficient.

The gamma absorbed dose contribution was investigated using the ISO-PC computer code (Rittmann, 2004). All requisite data for ^210^Po are provided by Rittmann (2004).

Given the small size of the microsphere and target arteriole wall, photon calculations are relatively insensitive to the model geometry (i.e., the microsphere imbedded in a cylindrical, slab, or a spherical shield). Accordingly, the calculations were performed using the concentric sphere option of the ISO-PC Code (IGEOM = 3).

### Relative biological effectiveness

The absorbed dose is often modified by the relative biological effectiveness (RBE) to account for the cell killing efficiency of a given radiation type. An RBE is usually defined as the ratio of a dose of reference energy photons to a dose of another radiation type that produces the same biological effect. Although the RBE is a relatively simple concept, its therapy application is complex (IAEA, 2008). This complexity arises because the RBE depends on the radiation type and its energy, the delivered absorbed dose, the delivery method (e.g., fractionization sequence), and the cell and tissue type that is irradiated. Given these uncertainties and the current stage of ^210^Po microsphere development, no RBE is applied to the absorbed doses calculated in this paper. Since the anticipated RBE values for alpha particles will be greater than unity, the absorbed dose effectiveness for tumor disruption provided in this paper represents a lower bound of the performance of the ^210^Po microsphere approach.

The proposed ^210^Po approach differs from current ^90^Y microsphere therapy in terms of the radiation type delivering the absorbed dose. For ^210^Po the absorbed dose is derived from alpha particles while for ^90^Y it is attributed to electrons. A consideration of typical relative biological effectiveness values suggests that the biological effective dose for alpha particles is about 20 times greater than electrons (IAEA, 2008; Bevelacqua, 2010a). This difference must be considered in the microsphere design in evaluating the absorbed dose delivered to the target vascular tissue as well as the dose that may be inadvertently delivered to healthy tissue. Dose delivery to healthy tissue (e.g., lung) is addressed in subsequent discussion.

## Results and Discussion

**Figure 1:**
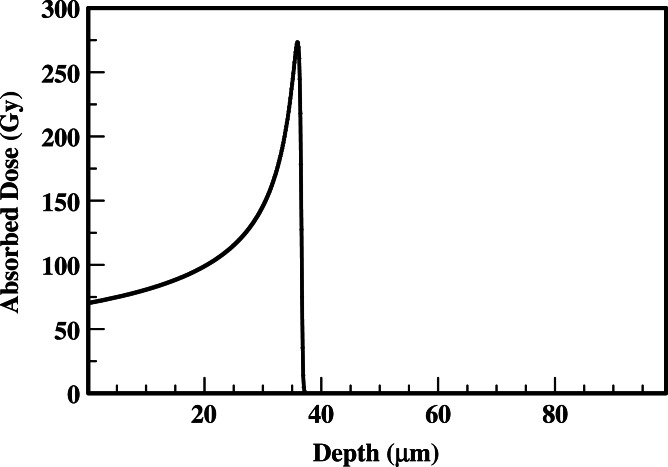
Absorbed dose profiles for ^210^Po alpha particles in water. The absorbed dose is delivered by a 1 µm diameter sphere following the total decay of 0.3 Bq uniformly deposited within the microsphere.

**Figure 2:**
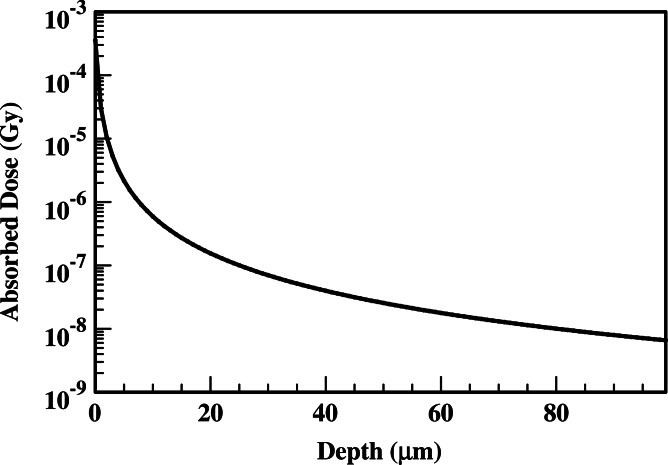
Absorbed dose profiles for ^210^Po photons in water. The absorbed dose is delivered by a 1 µm diameter sphere following the total decay of 0.3 Bq uniformly deposited within the microsphere.

In performing the absorbed dose calculations, the ^210^Po microsphere is assumed to reside at the inner arteriole wall. The results of Figs. 1 and 2 provide absorbed dose profiles for blood vessel wall thickness values ≤100 µm. Water is assumed to be the medium comprising the vessel wall which is a reasonable tissue surrogate for initial calculations (Kraft, 2000; Bevelacqua, 2005).

The results of the alpha and gamma contributions to the absorbed dose from a ^210^Po loaded microsphere are summarized in Figs. 1 and 2, respectively. The gamma absorbed dose is significantly less than the alpha absorbed dose. Therefore, Fig. 1 also represents the total absorbed dose delivered by the candidate ^210^Po microspheres.

Dose localization within the tumor vasculature is quite good with the peak dose delivered at 35.9 µm. At the Bragg peak, the ratio of alpha to gamma dose is 1.7 × 10^10^. Beyond 37 µm, less than 0.1 µGy is delivered by the ^210^Po photons.

Although the ^210^Po microsphere has good dose localization, its 138 d half-life is larger than currently used radionuclides ^32^P (14.28 d) and ^90^Y (2.669 d). As noted by Bevelacqua (2014), the complete design specifications will involve various methods to deliver and remove the microspheres from the body.

The delivered dose rate as a function of time is given by the relationship:

(5) \begin{eqnarray*} \displaystyle \dot {D}(t)=\dot {D}(0){\mathrm{e}}^{-\lambda t}&&\displaystyle \end{eqnarray*}

where *λ* is the ^210^Po physical decay constant. The total delivered dose to the arteriole wall at time T is obtained by integrating Eq. (5):

(6) \begin{eqnarray*} \displaystyle \int \nolimits \nolimits _{0}^{T}\dot {D}(t)d t=\int \nolimits \nolimits _{0}^{T}\dot {D}(0){\mathrm{e}}^{-\lambda t}d t&&\displaystyle \end{eqnarray*}

(7) \begin{eqnarray*} \displaystyle D(T)=\frac{\dot {D}(0)}{\lambda }(1-{\mathrm{e}}^{-\lambda T})=D(\infty )(1-{\mathrm{e}}^{-\lambda T}).&&\displaystyle \end{eqnarray*}

The ratio of *D*(*T*) to the maximum delivered dose *D*(∞) is:

(8) \begin{eqnarray*} \displaystyle \frac{D(T)}{D(\infty )}=(1-{\mathrm{e}}^{-\lambda T}).&&\displaystyle \end{eqnarray*}

Since the activity is proportional to the delivered dose, early removal of the microsphere at time *T* requires an increase in the initial activity loading by a factor *F*

(9) \begin{eqnarray*} \displaystyle F=\frac{1}{(1-{\mathrm{e}}^{-\lambda T})}.&&\displaystyle \end{eqnarray*}

The requisite increase in activity as a function of irradiating time (*T*) is illustrated in Table 4. This activity increase delivers the required dose to disrupt the microsphere as noted in Figs. 1 and 2. For example, an activity of about 2 Bq (0.3 Bq × 7.17) is the requisite ^210^Po activity loading to produce the doses summarized in Figs. 1 and 2 if the microspheres are removed at 30 d.

**Table 4:** ^210^Po microsphere activity increase required for early removal.

*T*(*d*)	*F* ^a^
10	20.5
30	7.17
50	4.51
70	3.38
100	2.54
300	1.29
∞	1.00

**Notes.**

a See Eq. (9).

## Delivery Methods

In the initial approach, it is likely that the microspheres will be directed into the tumor vasculature using a catheter. The catheter will likely enter through an artery or vein to reach the candidate tumor vasculature. Following the ^90^Y microsphere liver treatment delivery method, a catheter would be guided through the femoral artery into the liver and deliver the microspheres into the tumor’s vasculature. Image-guided radiation therapy (Lee & Seong, 2012; National Cancer Action Team, 2012) could be utilized to facilitate guiding the catheter to specifically target the tumor vasculature.

A number of tumors could be treated using the catheter delivery approach. For example, access through the renal artery would facilitate ^210^Po microsphere delivery to tumors residing in the kidneys. Other cancer locations (artery or vein access) include: lungs (pulmonary artery), head and neck (carotid artery), and stomach, intestines and liver (hepatic portal system). Arteries and veins connect with appropriate blood vessel subsystems to provide a microsphere pathway to the desired locations. The specific catheter path for the various tumor types will be refined and developed in a manner that was similar to the evolution of the ^90^Y microsphere treatment of liver cancers (Carmeliet & Jain, 2000; Kennedy & Salem, 2003; Carr, 2004; Murthy et al., 2005; Kennedy et al., 2007; Chaudhury et al., 2010; Rajput & Agrawal, 2010).

Accomplishing preferential blood vessel attachment requires significant development. For example, the microsphere design effort will evaluate a variety of chemical and physical approaches (e.g., electric charge, heat, pH, and electromagnetic fields) to facilitate the preferential attachment of the microsphere to the tumor’s vascular wall. The specific microsphere design characteristics that require experimental investigation include material composition, electric charge and its spatial distribution, physical size and shape, electromagnetic field strength, and dielectric and diamagnetic characteristics. Final microsphere design may be enhanced using activating agents, which could include the use of lasers, heat, electric fields, magnetic fields, and a spectrum of electromagnetic radiation. Both the microsphere design and activating agents require extensive verification efforts. These investigations and the results of this paper are required before a microsphere experiment can be performed. Accordingly, no experimental data associated with tumor vascular disruption are included in this paper.

## Removal Methods

The results of Table 4 require that a removal mechanism be developed to extract the microspheres at the prescribed time. The removal approach would in principle be accomplished by reversing the attachment protocol noted in a previous section. For example, one possible mechanism utilizes the inherent properties of magnetic materials. Placement and subsequent removal could be achieved by embedding a magnetic material (e.g., iron particles) within the ^12^C microsphere. The magnetic particles facilitate placement in the desired location using an active, localized magnetic field. Eliminating the magnetic field would facilitate microsphere removal. The protocol for microsphere implantation and removal require additional research and development.

### Possible enhancements

The ^210^Po microsphere modality could be expanded to a more global set of tumors by incorporating nanotechnology into the construction of a second-generation microsphere. Appropriate nanotechnology would permit the spheres to be guided to the location of interest following injection into the body. Upon reaching the desired location, the microspheres would be activated to either attach or disengage from the wall of the tumor’s vasculature. This second-generation technology does not currently exist and would be part of the process for developing and advancing the ^210^Po microsphere approach.

These two approaches require the prior detection of a tumor to initiate treatment. These tumors are well-established and are of sufficient size to permit detection. A significantly more advanced microsphere approach would involve the detection of tumors in their infancy and target the microspheres to the specific location of an infant tumor. This approach would utilize a smaller quantity of radioactive material since the tumors would be small and their vasculature structures more susceptible to attack.

## Practicality of the Approach

Vascular disruption using microspheres has been accomplished using ^32^P and ^90^Y (Kennedy & Salem, 2003; Carr, 2004; Murthy et al., 2005; Kennedy et al., 2007; Chaudhury et al., 2010; Rajput & Agrawal, 2010; Dezarn et al., 2011; Lau, Lai & Leung, 2011; Lee & Seong, 2012; Amor-Coarasa et al., 2014). As such the approach has been demonstrated by these authors to be feasible. Using a different isotope represents a minor adjustment to the methodology. As such implanting a ^210^Po microsphere and leaving it in the body represents a viable approach.

The capability of preferentially depositing microspheres in the liver has also been demonstrated (Kennedy & Salem, 2003; Carr, 2004; Murthy et al., 2005; Kennedy et al., 2007; Chaudhury et al., 2010; Rajput & Agrawal, 2010; Dezarn et al., 2011; Lau, Lai & Leung, 2011; Lee & Seong, 2012; Amor-Coarasa et al., 2014). Therefore, preferential deposition is likely to be achieved using catheter delivery. However, the approach proposed in this paper is more speculative because it proposes to selectively deposit and then remove the microspheres at a desired time. This proposal must be demonstrated experimentally. Since this paper strictly addresses the theoretical aspects of a proposed approach, demonstration of selective deposition and removal of microspheres is outside the scope of this paper.

An additional issue associated with ^210^Po microspheres is their physical half-life. ^210^Po and ^90^Y are very different in terms of their physical half-lives (*T_p_*) which must be addressed before the approach can be advanced as a viable treatment protocol. ^210^Po has a physical half-life of 138 days while ^90^Y’s half-life is only 64 h. The treatment delivery method used for ^90^Y(liver cancer for example) may not work for ^210^Po if the microsphere construction does not limit the biological half-life of the device. For example, some ^90^Y microspheres may flow with blood into the lung and remain in healthy tissue. For a relatively short physical half-life material like ^90^Y, this does not produce a significant absorbed dose. However, the longer physical half-life of ^210^Po would produce a larger residual lung dose that could create a significant biological detriment. This concern is alleviated if the ^210^Po microspheres are constructed of a material that has a short-biological half-life in the lung.

The capability to construct microspheres with a short lung retention time is a necessary design requirement for the final ^210^Po development effort. For example, the one micron microspheres will be constructed of a material with ICRP 30 (ICRP, 1979) Class D lung retention characteristics. As defined in ICRP 30, Class D material has a lung biological half-life that is less than 10 days. Part of the development effort will be the construction of a microsphere with a short biological retention time in the lung. Since the effective half-life (*T_e_*) of a radionuclide depends on its biological and physical half-lives, a longer physical half-life is not a limiting factor if the biological half-life (*T_b_*) is short. The effective half-life of the ^210^Po microsphere in the lung is given by the standard relationship (Bevelacqua, 2010b):

(10) \begin{eqnarray*} \displaystyle {T}_{e}=\frac{{T}_{p}{T}_{b}}{{T}_{p}+{T}_{b}}.&&\displaystyle \end{eqnarray*}

The ^210^Po microsphere effective half-life in the lung for a Class D material with 2 and 10 day biological half-lives is 1.97 and 9.32 days, respectively. A microsphere design requirement for an ICRP Class D level biological half-life in the lung eliminates the concern associated with the longer ^210^Po physical half-life.

## Safety Issues

^210^Po can be effectively utilized for vascular disruption if it is retained in the microsphere. However, if the ^210^Po leaches from the microsphere, it can be radiotoxic to the patient, treatment team, and the public. Since ^210^Po is a very high activity radioactive isotope, it merits careful handling to ensure safety of the patient, treatment team, and the public. This section addresses these radiological aspects in order to ensure that ^210^Po can be used in a safe manner

The following discussion presupposes that the developed microspheres have high ^210^Po retention capability. This is a necessary design requirement since ^210^Po is radiotoxic. The capability of handling highly radiotoxic materials is well known and materials with similar alpha radiation types (e.g., ^239^Pu and ^241^Am) can be safely addressed (Bevelacqua, 2010b) using good radiological controls practices.

### ^210^Po toxicity and patient safety

^210^Po has a specific activity of 1.7 × 10^14^ Bq/g and its inhalation (ingestion) effective dose coefficient is 3.0 × 10^−6^ Sv/Bq (2.4 × 10^−7^ Sv/Bq) (ICRP, 2012). The microsphere intake pathway caused by leaching has not been evaluated, but its effective dose coefficient is probably in the range of the established conventional intake pathway values, which is approximately 10^−6^ Sv/Bq. For the proposed base case microsphere of 0.3 Bq, complete ^210^Po leaching from a single microsphere would lead to an effective dose of about 0.3 µSv. Since the number of microspheres would be utilized in a therapy protocol, the effective dose from complete leakage is limited only if the microspheres have good retention characteristics. However, the final microsphere design should have good ^210^Po retention characteristics. With good ^210^Po retention characteristics, the toxicity hazard to the patient is not significant. For example, if 10^6^ 0.3 Bq microspheres were administered with a ^210^Po retention of 90%, the patient 50 year effective dose commitment is only 30 mSv.

### Safety of the treatment team

The treatment team administers the ^210^Po material and could become contaminated during transfer of the microspheres to the patient. A medical physicist should verify that the correct treatment activity is contained in the delivery device. This should occur prior to transport from the Nuclear Pharmacy to the treatment area.

The medical physicist with radiological controls technicians support transport the delivery vial, the transfer container in an appropriate shield, and radiation detectors to the treatment room. All treatment team members wear protective clothing including gloves, lab coat, shoe covers, surgical cap, and a face mask to minimize personnel contamination.

During the procedure, radiological controls technicians monitor personnel and equipment for residual ^210^Po contamination. Any contamination control problems with the administration are indicated by positive radiation detector readings. Contamination is controlled upon detection and measures are taken to minimize the intake of radioactive materials by the patient and staff.

After the procedure, the medical physicist monitors all the staff and the patient for contamination. Following personnel monitoring, the administration table, delivery equipment, and the floor are monitored for contamination. All contaminated areas are immediately decontaminated by the radiological controls technicians.

The technicians return the waste container, used delivery vial, transport containers, and the radiation detectors to the Nuclear Pharmacy. The medical physicist and pharmacy verify that the correct activity has been delivered. All materials are properly stored or disposed as radioactive waste.

### Safety during transportation and storage

Radioactive materials must be placed in a secure location during storage. The storage area should be restricted to qualified radiological controls personnel assigned to the treatment team. For ^210^Po these materials are located in a secure locked cabinet with keys maintained by the facility radiation safety officer. Prior to placing the materials into storage, all containers must be free of residual contamination. Radiation monitoring must also verify all ^210^Po containers are also free of alpha contamination.

After the containers are free of external contamination, they should be placed in a transport container for movement to the treatment area. Hospital security staff should clear the transport route of all visitors and hospital staff to facilitate safe transport and minimize the probability of accidents that could disperse the material. Upon reaching the treatment area, radiological controls personnel deliver the ^210^Po containers to the treatment staff for administration to the patient. Each step of the transfer process is verified by radiation monitoring to ensure that no residual ^210^Po has been released into areas not under the control of qualified radiological personnel.

### Safety of the public

Public safety is ensured by minimizing the likelihood of ^210^Po escaping from the treatment area. Good radiological controls practices restrict the movement of any residual ^210^Po activity. These controls are implemented by radiological controls technicians and professionals assigned to the treatment team.

As with all radiological controls practices, the radioactive material must be controlled within the area of its use. Following administration, all vials and transport containers are surveyed for residual alpha contamination. Any contaminated materials should be decontaminated. Decontamination waste is bagged in plastic and labeled for proper disposal. If decontamination is unsuccessful, the material is packaged for further disposition by qualified radiological controls personnel.

All treatment personnel are periodically monitored for contamination. Any contaminated materials are retained for proper disposal. Personnel must also be thoroughly monitored for contamination before exiting the treatment area. By carefully monitoring personnel and equipment, radioactive material is retained within the treatment area and not permitted to enter areas occupied by the public. The use of hand-held radiation instruments sensitive to alpha radiation and alarming personnel contamination monitors facilitate this effort. Alarming exit portal monitors provide an additional measure of safety to protect the public.

## Prospective and Significance

The proposed vascular disruption approach is currently a theoretical endeavor. Ideally, it would have an experimental basis, but that is beyond the scope of this paper. Future experimental effort will be based on the calculations of this paper as well as development of a detailed microsphere design. For example, the capability of ^210^Po microspheres to preferentially attach to a tumor’s blood vessels and avoid the issues associated with transporting radioactive material to other tissues must be demonstrated.

## Conclusions

The ^210^Po microspheres outlined in this paper provide good dose localization that deposits the requisite absorbed dose for vascular disruption to depths of about 37 µm with minimal dose beyond that location. Elimination of absorbed dose to healthy tissues minimizes any radiologically related side effects. Although in the theoretical development phase, ^210^Po microsphere vascular disruption therapy offers the potential for a protocol that preferentially deposits absorbed dose in the arteriole wall.
